# Intravascular Germ Cell Tumor With Cardiac and Bilateral Pulmonary Artery Involvement

**DOI:** 10.1016/j.jaccas.2025.103319

**Published:** 2025-04-09

**Authors:** Camilo Andres Calderon-Miranda, Maria Juliana Reyes-Cardona, Gabriel Roberto Lopez-Mora, Eduardo Alberto Cadavid-Alvear, Carlos Enrique Vesga-Reyes, Jairo Sanchez-Blanco, Pastor Olaya, Jorge Alexander Zambrano-Franco

**Affiliations:** a Departamento de Cardiología, Fundación Valle del Lili, Cali, Colombia; b Facultad de Ciencias de la Salud, Universidad Icesi, Cali, Colombia; c Centro de Investigaciones Clínicas, Fundación Valle del Lili, Cali, Colombia; d Departamento de Medicina Interna, Fundación Valle del Lili, Cali, Colombia; e Departamento de Cirugía Cardiovascular, Fundación Valle del Lili, Cali, Colombia

**Keywords:** cancer, computed tomography, echocardiography, pediatric surgery

## Abstract

**Background:**

Germ cell tumors (GCTs) are neoplasms usually originating in the gonads, most often affecting young patients. Intravascular GCTs are exceptionally rare and potentially fatal due to cardiac and embolic complications.

**Case Summary:**

A 17-year-old patient presented with weight loss, abdominal pain, and dyspnea. Imaging revealed an intravascular mass extending from the inferior vena cava (IVC) into the right heart chambers and pulmonary arteries. The tumor was successfully removed via surgery under cardiopulmonary bypass. Histopathologic diagnosis was a mixed non-seminomatous GCT. Recovery was uneventful, and follow-up imaging showed no intravascular lesions. Cisplatin-based chemotherapy was started with curative intent.

**Discussion:**

Intravascular GCT is a rare phenomenon, presenting mostly with retroperitoneal disease affecting the IVC. Cardiologists assess complications and interpret cardiovascular imaging. A multidisciplinary approach is crucial for improving outcomes.

## Visual Summary

**Figure:**
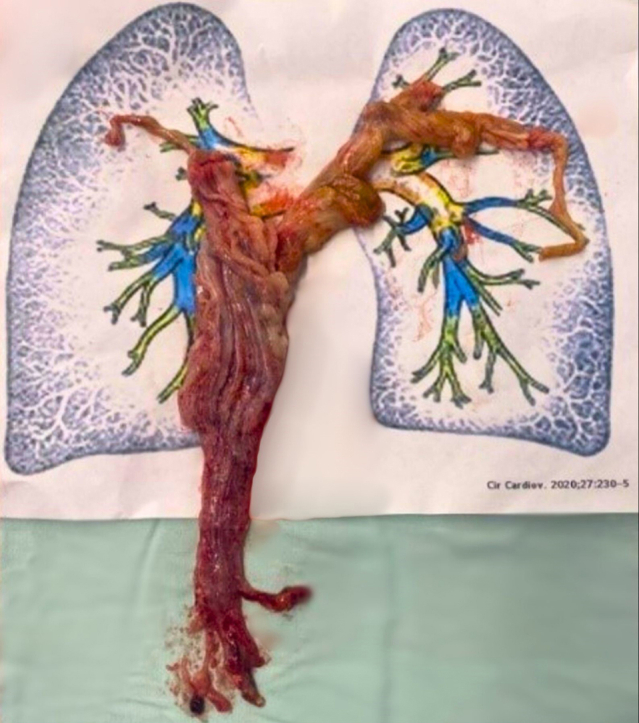

## History of Presentation

A 17-year-old patient presented with a 4-month history of weight loss of 18 kg, diffuse abdominal pain of moderate intensity, palpitations, and progressive dyspnea on exertion. An extra-institutional transthoracic echocardiogram revealed an intracavitary mass extending from the inferior vena cava (IVC) to the right heart chambers. The patient was transferred to our high complexity institution for further studies. Upon admission, vital signs were within normal range, cardiac sounds were rhythmic and regular without murmurs, lungs were clear to auscultation, there was abdominal pain on palpation, and there was right testicular edema without testicular masses.

**Take-Home Messages**

• Intravascular GCTs are rare entities with a high risk of embolic and cardiac complications, requiring multidisciplinary evaluation and treatment.
• Cardiovascular imaging holds an essential role in the characterization and preoperative planning of intravascular masses.

## Past Medical History

The patient had no past medical history. Immunizations were up to date.

## Differential Diagnosis

The progressive onset of symptoms, significant weight loss, abdominal pain, and imaging suggesting an intravascular mass raised concern for a malignancy with extension to the IVC, including renal cell, hepatocellular, and adrenocortical carcinoma. Extensive IVC thrombosis was also considered.

## Investigations

Initial laboratory testing included hemoglobin 10.5 g/dL, leukocyte count 12,390/μL, platelet count 537,000/μL, creatinine 0.77 mg/dL, beta-human chorionic gonadotropin 247 mIU/mL, alfa-fetoprotein 99 ng/mL, and lactic acid dehydrogenase 555 U/L.

A transthoracic echocardiogram revealed normal left ventricular systolic and diastolic function (left ventricular ejection fraction of 57%), mild dilation of the right ventricle with normal contractile function (fractional area change of 35%), and a large, highly mobile image with intermediate echogenicity passing through the right chambers without compromising the pulmonary or tricuspid valve closure mechanism (Videos 1 to 3).

**Figure 1:**
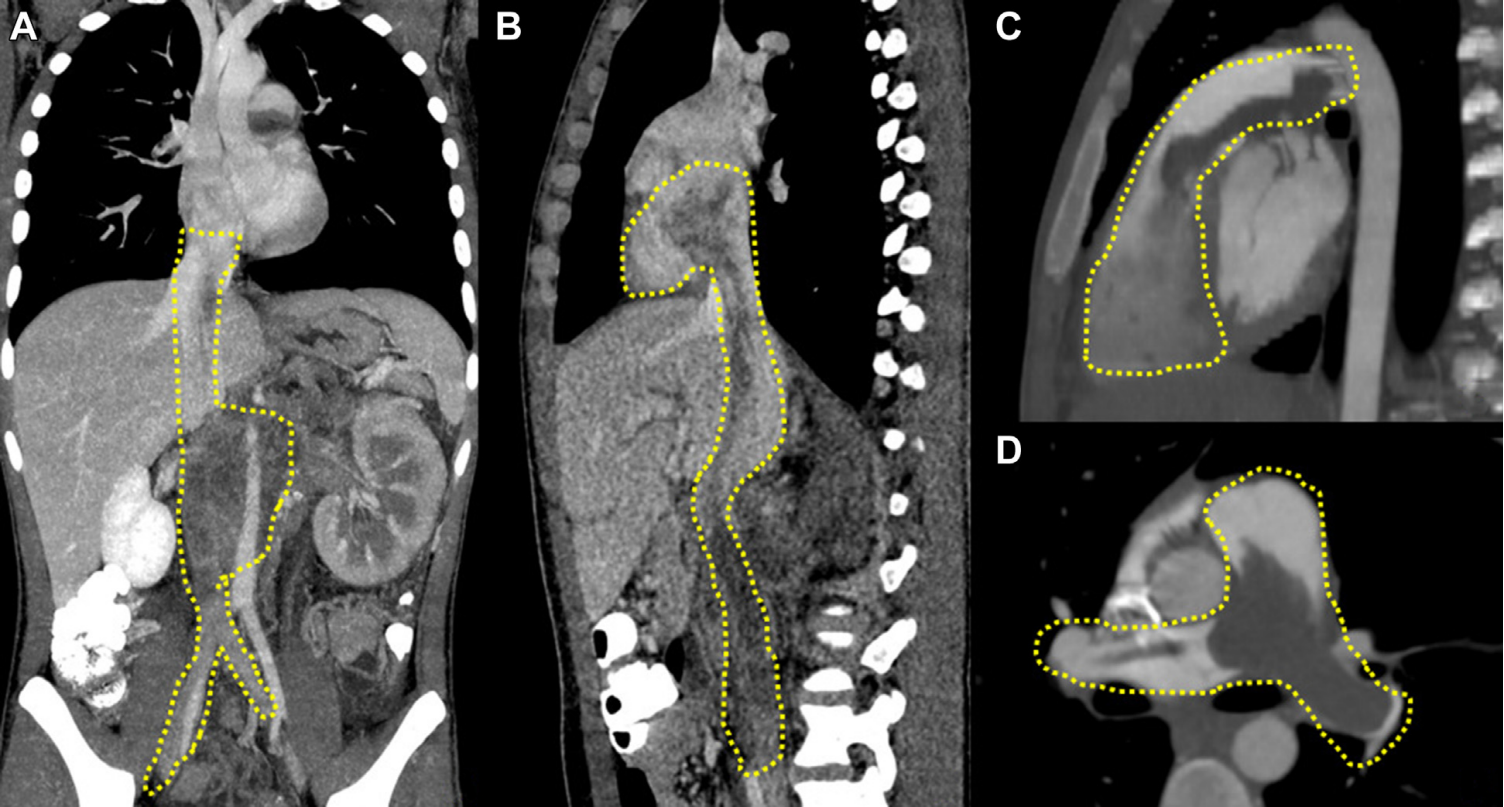
Thoracoabdominal CT Angiography The yellow dotted lines outline the vascular structures. (A) Coronal plane. Retroperitoneal tumor infiltrating the inferior vena cava and extending to the intrahepatic portion of the vena cava. (B) Oblique sagittal reconstruction. Extension of the tumor toward the right atrium. (C) Oblique sagittal reconstruction. Extension of the tumor toward the right ventricle and pulmonary trunk. (D) Axial plane. Extension of the tumor toward the pulmonary arteries. CT = computed tomography.

**Figure 2:**
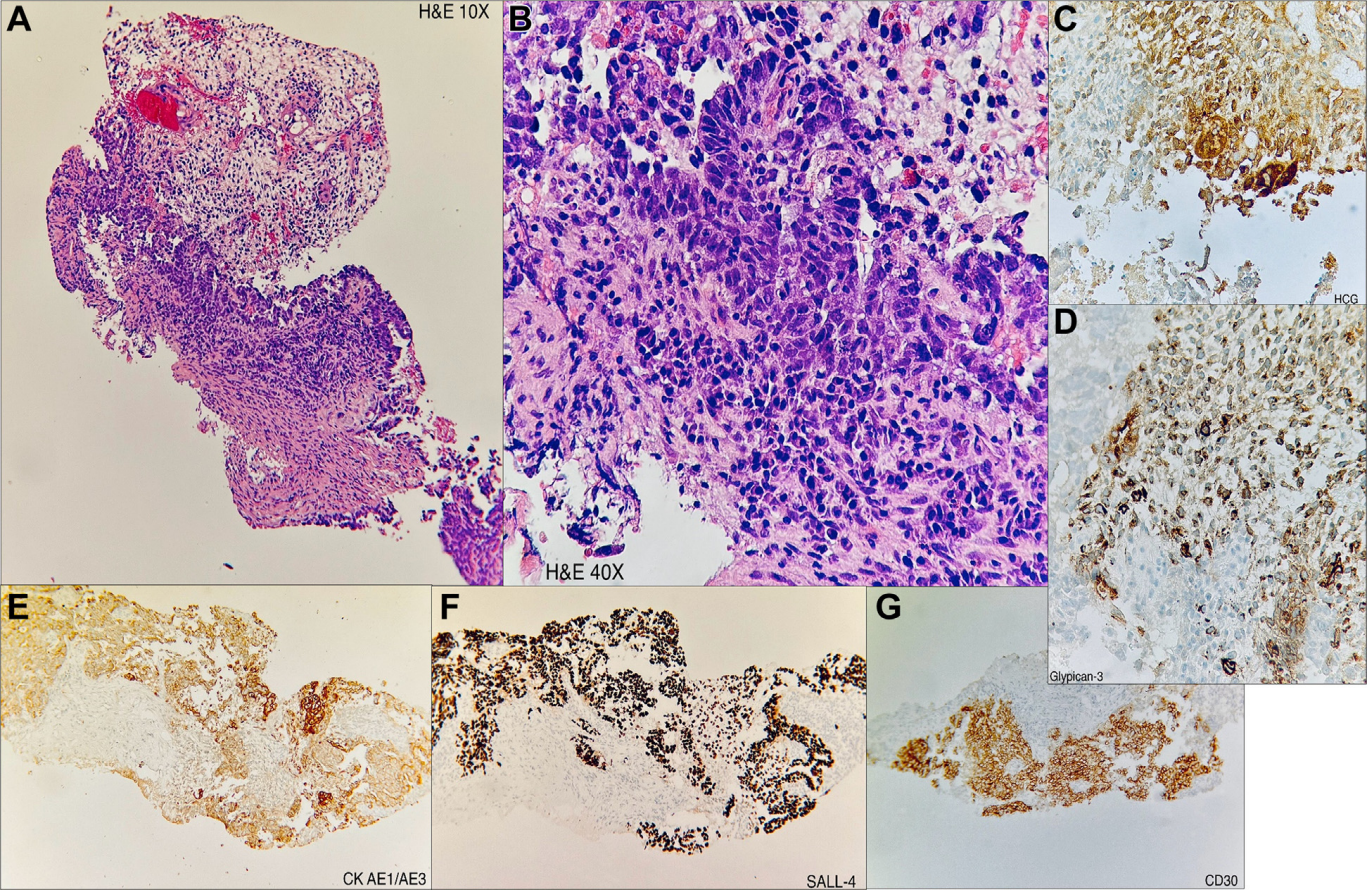
Intra-Abdominal Mass Percutaneous Biopsy Histologic and immunohistochemical stains suggest the diagnosis of a mixed non-seminomatous germ cell tumor. (A) Hematoxylin and eosin (H&E), 10×: overview of the tissue architecture. (B) H&E, 40×: pleomorphic cells, increased nuclear-to-cytoplasmic ratio. (C) Human chorionic gonadotropin (HCG) stain is positive, suggesting HCG-producing cells. (D) Positive glypican-3 stain. (E) Positive cytokeratin AE1/AE3 (CK AE1/AE3), indicating epithelial origin. (F) SALL4 stain showing strong nuclear positivity. (G) CD30 stain, positive in specific cell clusters.

Thoracoabdominal computed tomography (CT) angiography revealed a large tumor thrombus, which involved the IVC from its origin, passing through the right atrium and ventricle and extending to the main pulmonary artery, bilaterally. Multiple pulmonary nodules were detected bilaterally. In addition, extensive retroperitoneal lymph node involvement was observed (Figure 1). A CT-guided biopsy of the intra-abdominal tumor was performed, with histopathology findings suggesting a diagnosis of a mixed non-seminomatous germ cell tumor (GCT) with predominance of embryonic carcinoma, with a yolk sac tumor component and presence of syncytiotrophoblast cells (Figure 2). Testicular ultrasound was performed to exclude a primary gonadal GCT, without evidence of solid masses.

## Management

A multidisciplinary meeting with the oncology, cardiology, cardiovascular surgery, and radiology teams took place to decide on a treatment strategy with curative intent. There was a high risk of tumor lysis syndrome and embolism of tumor fragments upon start of chemotherapy due to the large tumor burden and expected high sensitivity to a cisplatin-based regimen; it was therefore decided to first perform surgical extraction of the mass. Surgical intervention (Video 4) was conducted under cardiopulmonary bypass with anterograde cerebral perfusion. The tumor was extracted from 2 anatomical sites: the right atrium and a 3 cm incision in the pulmonary artery. During the period of cardiac arrest, incision of the structures and traction of the tumor with constant moderate force were performed. From the right atrial incision, the intraluminal IVC and intracardiac tumor were removed, and from the pulmonary artery, the tumor extending to the pulmonary branches was extracted. There was no significant bleeding; the described incisions were closed with continuous polypropylene suture. A 50-cm mass was obtained.

**Visual Summary:**
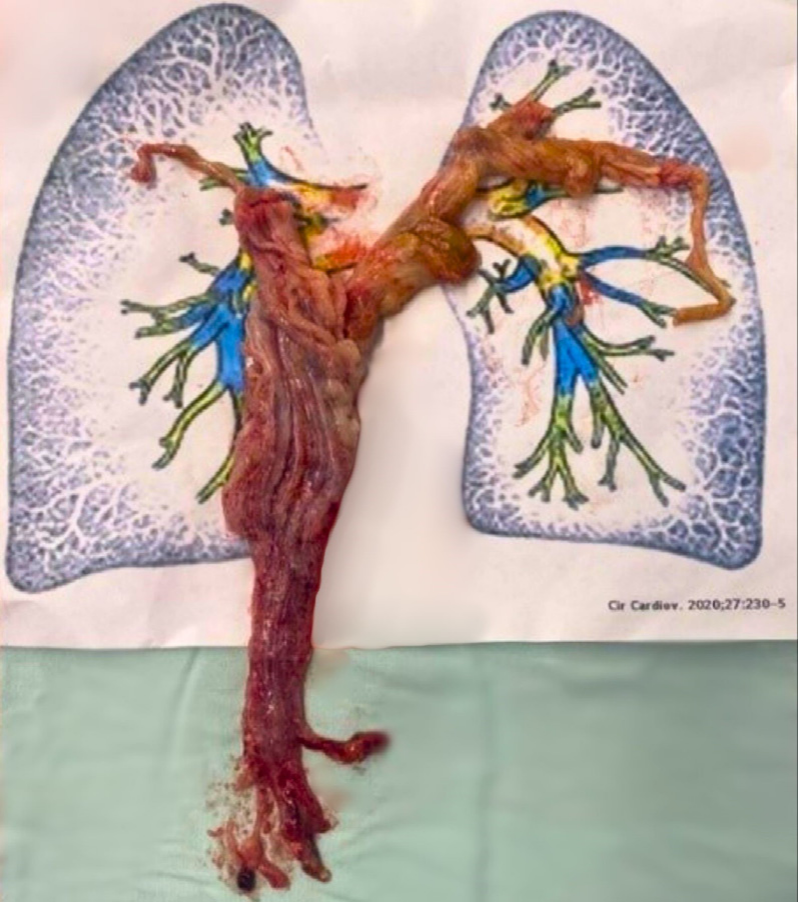
Surgical Specimen A 50 cm intravascular mass extending from the inferior vena cava to the right heart chambers and pulmonary arteries.

## Outcome and Follow-Up

Postoperative evolution was satisfactory; the patient was weaned from mechanical ventilation and vasopressor support in the first 24 hours. A control CT angiography ruled out residual intravascular lesions, excluding the need for an IVC filter. Surgical specimen pathology confirmed previous findings. Considering the diagnosis of a stage IIIB nonseminomatous mixed GCT with International Germ Cell Cancer Collaborative Group classification of “good risk,” 13 days after the surgical procedure, clinical oncology started the patient on adjuvant chemotherapy with a bleomycin, etoposide, and cisplatin (BEP) protocol. He received 2 cycles with adequate tolerance and was discharged for outpatient oncology follow-up and continuation of the chemotherapy regimen in another institution in his city of origin.

## Discussion

Extragonadal GTCs (EGCTs) are rare neoplastic tumors thought to arise from errors in midline migration of germ cell precursors or metastases from occult or regressed primary gonadal tumors. It has been proposed that retroperitoneal EGCTs nearly always originate from a primary testicular tumor. EGCTs encompass seminomatous tumors, including only classical seminoma, and non-seminomatous tumors, which consist of embryonal carcinoma, choriocarcinoma, yolk sac carcinoma, mature or immature teratoma, and mixed GCTs constituted by any combination of ≥2 GCT histotypes.^1^ The most frequent extragonadal location is mediastinal, comprising 50% to 70% of EGCTs, followed by retroperitoneal (30%-40%) and intracranial tumors.^2^ Due to the finding of large retroperitoneal conglomerates with intravascular dissemination and absence of testicular solid masses, our case was consistent with a primary retroperitoneal EGCT. Laboratory studies include serum lactate dehydrogenase, which is an unspecific tumor mass marker elevated in 40% to 60% of all GCTs, and the specific tumoral markers alpha-fetoprotein and beta-human chorionic gonadotropin, with elevation of either or both occurring in approximately 80% of non-seminomas^3^ (including the current case).

Intravascular GCT thrombus is a rare phenomenon, presenting mostly synchronous and in the context of advanced primary gonadal disease with retroperitoneal disease affecting the IVC.^4^, ^5^, ^6^ In this paper, we report a case of EGCT with extensive intravascular involvement extending from the IVC through right cardiac chambers to the pulmonary arteries bilaterally and reaching the secondary branches of both pulmonary trees; only a limited number of cases have been reported in the medical literature of this scenario.

Intravascular tumors pose a high risk of embolization to pulmonary circulation, paradoxical embolization in the presence of shunts, valvular involvement, hemodynamic repercussions due to cardiac occupation, and secondary hematologic involvement, including thrombocytopenia and hemolytic anemia.^5^ Patients with intracardiac tumors may present with chest pain, cough, dyspnea, syncope, new murmurs, and right ventricular failure. Pulmonary thromboembolism manifestations include dyspnea and hemoptysis; even cardiopulmonary arrest in the context of pulmonary thromboembolism secondary to intravascular GCTs has been reported.^7^

Imaging is key in the diagnosis of intravascular masses and their complications, as in preoperative planning. CT angiography allows for characterization of intravascular findings, and echocardiography is useful for evaluation of intracavitary occupation, description of the size and location of the mass, exclusion of paradoxical embolization risk, and assessment of cardiac function, including valvular involvement.^8^

The cardiologist plays a central role in the clinical approach, diagnosis, and management of intravascular and cardiac masses, often being the first to evaluate symptoms such as shortness of breath, chest pain, embolic events, or heart failure. As stated in the *JACC: CardioOncology* State-of-the-Art Review of cardiac tumors,^8^ the diagnostic approach for a cardiac mass must take into account the age of the patient, clinical probability, location of the tumor, and cardiovascular magnetic resonance tissue characterization. The finding of an intraluminal mass in the IVC extending to the right cardiac chambers must raise suspicion for renal cell carcinoma, corresponding to 85% of intraluminal IVC tumors, followed by hepatocellular carcinoma, adrenal tumors, retroperitoneal sarcomas, and, less frequently, primary IVC leiomyosarcoma. Despite its low incidence, an intravascular GCT is a relevant differential diagnosis as it affects a younger age group, and its generally high chemosensitivity allows for a curative intent in most cases, even in metastatic disease.^9^

Management of this tumor must be multidisciplinary. The risk of cardiac and embolic complications and need to reduce tumor load before starting systemic therapy generally warrant surgical indication. Cardiac and pulmonary involvement in this case led to the need for cardiopulmonary bypass for the extraction of the tumor. The procedure was successful without residual masses in subsequent imaging, and chemotherapy was started without complications.

The Spanish Society of Medical Oncology and Spanish Germ Cell Cancer Group clinical guidelines for the management of germ-cell testicular cancer (2023)^10^ recommend using the International Germ Cell Cancer Collaborative Group prognostic classification. They state the standard therapy for patients with good prognosis is 3 cycles of chemotherapy with bleomycin, etoposide, and cisplatin (BEP); these recommendations are considered valid for extragonadal retroperitoneal and mediastinal tumors. The treatment planned for our patient followed this approach without complications during the first 2 cycles.

The prognosis of a retroperitoneal EGCT is similar to that of metastatic testicular GCTs and better than mediastinal EGCTs. Good risk disease treated with chemotherapy BEP had a projected 2-year progression-free survival of 90% in the trial by De Wit et al.^9^

## Conclusions

Intravascular GCT is a rare phenomenon with a high risk of embolic complications and symptoms due to cardiac intracavitary occupation and valvular involvement. Our case illustrates a retroperitoneal EGCT with extensive intravascular involvement, successfully extracted with surgery under cardiopulmonary bypass. This entity carries a high morbidity and mortality load, which can be modified by timely diagnosis and combined surgical and medical management. Cardiovascular imaging holds an essential role in the characterization and preoperative planning of intravascular CGTs.

## Funding Support and Author Disclosures

The authors have reported that they have no relationships relevant to the contents of this paper to disclose.

## Appendix

Video 1 Transthoracic Echocardiogram (A) Parasternal long-axis view. A mass is observed in the right ventricle. (B) Parasternal right ventricular inflow view. Isoechoic mass with irregular borders crossing the tricuspid valve. (C) Apical 4-chamber view. Mild right ventricular dilation with preserved systolic function. Large mobile mass of friable appearance in the right chambers. (D) Subcostal view. Intravascular mass extending from the inferior vena cava to the right atrium

Video 2 Transthoracic Echocardiogram Parasternal short-axis, aortic valve level view. Passage of the mass through the tricuspid valve (A), heading to the right ventricle outflow tract (B), and protruding through the pulmonary valve to the pulmonary trunk (C)

Video 3 Transthoracic Echocardiogram Parasternal short-axis, aortic valve level view. Color Doppler imaging showing no evidence of significant tricuspid (A) or pulmonary (B) stenosis or insufficiency. (C) Subcostal view: Color Doppler showing non-turbulent flow in the inferior vena cava. (D) Continuous wave spectral Doppler of the pulmonary valve not suggestive of stenosis or insufficiency

Video 4 Surgical Procedure Under Cardiopulmonary Bypass and Sternotomy Right atriotomy and release of the inferior vena cava tourniquet were performed to remove the intracaval and cardiac tumor. The pulmonary trunk and main arteries were subsequently opened to remove the pulmonary portion of the tumor. The incisions were closed, the aortic clamp was released, and extracorporeal circulation was gradually withdrawn
